# A Biotechnological Platform Based on the Newly Isolated *Streptococcus thermophilus D4* to Obtain Viable Biomass and Exopolysaccharides for Enterocytes Wound Healing

**DOI:** 10.1002/bit.70063

**Published:** 2025-09-15

**Authors:** Alberto Alfano, Darshankumar Parecha, Sergio D'ambrosio, Maria d'Agostino, Donatella Cimini, Chiara Schiraldi

**Affiliations:** ^1^ Department of Experimental Medicine University of Campania Luigi Vanvitelli Napoli Campania Italy; ^2^ Microbial Biotechnology Lab University of Insubria Varese Lombardia Italy; ^3^ Department of Environmental, Biological and Pharmaceutical Sciences and Technologies University of Campania Luigi Vanvitelli Caserta Campania Italy

**Keywords:** biotechnological production, design of experiment (DoE), exopolysaccharides, Plackett–Burman screening, probiotic strain, wound healing

## Abstract

There is growing interest in newly isolated lactic acid bacteria from natural sources and traditional food manufacturing and transformation. *Streptococcus thermophilus* is able to improve the safety of dairy products, showing antioxidant and antimicrobial properties and also to produce bioactive molecules, such as exopolysaccharides (EPSs), usable in foods applications. An integrated bioprocess development was performed using *S.thermophilus* D4, for the production of viable biomass and EPSs using a media optimized through design of experiments. Plackett–Burman screening method was used twice to optimize biomass and exopolysaccharide production. Different fermentation strategies (batch and fed‐batch) were performed to increase yield and productivity. Fed‐batch processes increased biomass production approximately 2.5‐ and 7.5‐fold than batch processes with optimized and M17 media, respectively. Furthermore, a significant increase of 1.7‐ and 6‐fold EPS biosynthesis was observed. Finally downstream processes based on membranes were conducted to purify bioactive molecules (EPSs) used on Caco‐2 cells showing a significant effect in accelerating wound healing. Finally, purification and recovery of EPSs tested on Caco‐2 cells. The results obtained confirm the potential of newly defined medium to replace M17, which has animal‐derived nitrogen sources, for probiotic biomass and EPSs production with many applications in different fields.

## Graphical abstract

Optimizing media for potential probiotic *Streptococcus thermophilus* biomass and exopolysaccharide production was achieved using design of experiments. This was followed by process development in bioreactors, employing fed‐batch strategies, to increase the production of exopolysaccharide, to test it, after membranes purification, on cells proving the ability to increase tissue repair.

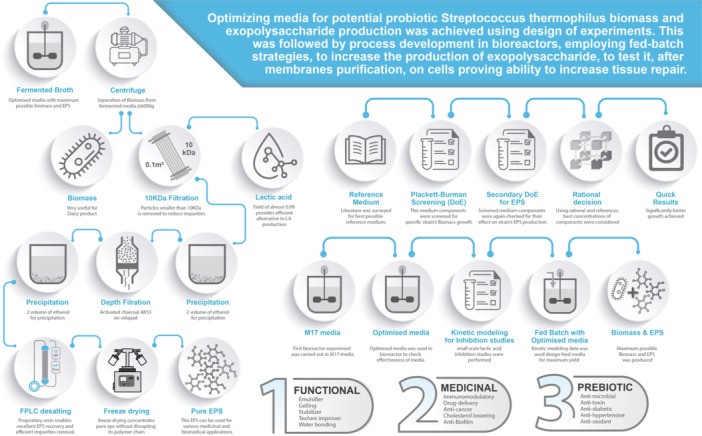

## 1 Introduction

*Streptococcus thermophilus* strains are among the most important commercial starter cultures used in dairy and artisanal industries for the production of cheese and fermented milk (Cui et al. 2016). These species can be used alone or in combination with other lactic acid bacteria (LAB) to produce dairy products such as cheese, yogurt, and fermented milk. Some previous studies have examined the fermentation role of S. thermophilus in improving safety of these dairy products (Han et al. 2022). In fact, most LAB may be recognized as probiotics because they can deliver multiple health benefits to the host if administered in adequate quantities through various ways (Tenea and Suárez 2020). Many studies report the probiotic potential of *S. thermophilus*, including antioxidant and antimicrobial activities, as well as modulation of intestinal microbiota (Iyer et al. 2010). Some strains have been shown to produce bioactive molecules, such as exopolysaccharides (EPSs), which have applications in functional foods and potential antitumor activity (Sun et al. 2018; Sørensen et al. 2022). EPSs produced by *S. thermophilus* are considered safe and are widely used to improve the rheology, viscosity, and consistency of fermented food products (De Vuyst et al. 2011). Their production ranges from 20 to 600 mg/L in milk‐based medium under optimal conditions (Mizuno et al. 2020). In particular, EPSs produced by *S. thermophilus* are often utilized by the dairy industry to enhance the rheological and physical properties of yogurt (Pachekrepapol et al. 2017; Linares et al. 2016).

Furthermore, EPSs derived from *S. thermophilus* have been increasingly studied for their diverse biological activities, making them a promise candidate for pharmaceutical and biomedical applications. Recent research highlights the antioxidant, antitumor, and periodontal regeneration properties of EPSs from *S. thermophilus* (Khalil et al. 2022).

EPSs production usually requires complex media, where carbon and nitrogen sources significantly influence cell growth and the synthesis of metabolic compounds. Additionally, factors such as temperature and pH need to be carefully controlled (De Vuyst et al. 1996). To meet economic constraints for industrial applications, media optimized for microorganism growth and EPS production have been developed using Design of Experiment (DoE) approaches. Optimized media for specific strains can increase biomass and bioactive molecule yields (Oleksy‐Sobczak and Klewicka 2020). The optimized media are then used in different fermentation strategies (batch and fed‐batch) to better understand the optimal nutrient combinations and process parameters, thereby increasing yield and productivity. A typical DoE experiment to optimize strain‐specific media involves screening various carbon and nitrogen sources along with salts, followed by fine‐tuning component concentrations to reduce the number of experiments needed. Nisha Suryawanshi and colleagues have reported media optimization studies using response surface methodology (RSM) and artificial neural networks (ANN) to enhance secondary metabolite production (Nisha Suryawanshi et al. 2019). These methods often require many experimental runs, resources, and time. Alternatively, screening methods such as Plackett–Burman, Taguchi, or OFAT can reduce the number of experiments while still providing accurate results. Recently, vegan‐grade media—free from animal‐sourced components—are gaining importance, as they reduce the carbon footprint and are as effective as traditional complex media (Parecha et al. 2024). By applying these methods, it is possible to optimize media for higher biomass or EPS It is experimentally noted that higher biomass may not necessarily result in higher EPS (Supramani et al. 2023). Therefore, it is advisable to develop a media to increase biomass, followed by screening experiments to optimize EPS production, also exploiting high‐cell density fermentation technologies (Subramaniam et al. 2018). The use of *S. thermophilus* D4 as a starter culture strain, combining fermentation and purification strategies, can be considered as “defining the product design space” of the concept of Quality by Design (QbD) (Rathore 2016). On other hand, defining the moment at which lactic acid removal shall initiate though detailed understanding of lactic acid inhibition kinetics should be considered as “defining process control strategy.” There are few research activities reported with the aim of defining the design space of probiotic bioprocess development, covering the optimization of culture media, fermentation method, and exopolysaccharide purification strategy (Rathore and Kapoor 2016). Furthermore, a recent study on *S. thermophilus* revealed its capacity to protect human intestinal epithelial cells (HT‐29) from oxidative damage induced by hydrogen peroxide. (Zhenxiang Xu et al. 2023). In addition, EPS and cellular components of *S. thermophilus* have been found to strengthen the intestinal barrier. Beyond barrier protection, recent studies highlight a role for *S. thermophilus*‐EPS in promoting intestinal epithelial wound healing. In particular, EPS has been found to accelerate wound closure in scratch assays using intestinal epithelial cells, suggesting a direct role in stimulating epithelial restitution (Chen et al. 2019). The aim of the work was to study different fermentation methods to identify the best solution in terms of biomass and exopolysaccharides production to be used in the nutraceutical and food fields, starting from a microorganism isolated from artisanal and local cheeses production. In the fermentation process, the production of biomass, EPS (exopolysaccharides), and lactic acid is often interconnected, but the relationships between these factors can vary depending on the microorganism, type and amount of substrate used and growth conditions (Fuso et al. 2023). Generally, an increase in biomass can favour a greater synthesis of EPS and lactic acid, even if this selection is not always direct nor linear. The availability of substrate significantly influences both microbial growth and metabolite production. For this reason, our aim was to optimize a fed‐batch fermentation process, defining an adequate feeding profile that avoids both overflow metabolism phenomena and substrate deficiencies, both factors potentially effective in inhibiting microbial growth and the resulting EPS synthesis (Sørensen et al. 2022). Particular attention was also paid to the production of lactic acid, a metabolite of interest for various industrial applications, but which, at high concentrations, can inhibit cell growth. To this end, lactic acid inhibition studies were conducted on the *S. thermophilus* D4 strain, with the aim of determining the maximum tolerable concentration for the microorganism.

Finally, exopolysaccharides produced by *S. thermophilus* were used on Caco‐2 cells for the wound healing assay.

## 2 Experimental Procedures

### 2.1 Bacterial Strain and Media Components

*S. thermophilus* D4, used in the study, was previously isolated from buffalo natural whey starters cultures for the production of artisanal cheeses. M17 medium used as reference was bought from Millipore, Switzerland. The nitrogen source used were neutralized soy peptone, guar peptone, and yeast extract (Organotechnie, France). Salts used were di‐sodium hydrogen phosphate (Na_2_HPO_4_) (Applichem, Germany), sodium dihydrogen phosphate (NaH_2_PO_4_) (Carlo Erba reagents, France), di‐sodium glycerophosphate (C_3_H_7_Na_2_O_6_P) (AppliChem, Germany), magnesium sulphate (MgSO_4_) (Biochem Chemopharma, France), manganese sulphate (MnSO_4_) (AppliChem, Germany), ferrous sulphate (FeSO_4_) (Biochem Chemopharma, France), and ascorbic acid (AppliChem, Germany). Sugar used were glucose, lactose, sucrose, maltose, and galactose (Sigma Aldrich, Italy).

### 2.2 Small Scale Experiments for Screening of Medium Components With DoE

The experiments were performed in 50 mL sterile Falcon tubes with 40 mL of medium, to leave just a low head air volume. Fermentation was carried out at 42°C with stirring of 150 rpm for 24 h in a shaker incubator (LAB Companion, WVR, Italy). The effects of various medium components and their concentrations on biomass production of *S. thermophilus* D4 were evaluated using medium compositions explained in Table 1A. All experiments were performed in duplicates. The optimized medium compositions were compared with reference medium M17 for the validation. Another set of DoE experiments using Plackett–Burman screening (PBS) was used to optimize EPS production. The media compositions are explained in Table 1B.

**Table 1A:** Medium component screening for Biomass—various factor setting value.

Factors	Code	Setting value		
		—	0	+
Glucose	A	0	10	20
Sucrose	B	0	10	20
Maltose	C	0	10	20
Galactose	D	0	10	20
Soya peptone	E	0	10	20
Guar peptone	F	0	10	20
Yeast extract	G	0	7.5	15
Ascorbic acid	H	0	0.25	0.5
MgSO_4_	I	0	0.25	0.125
MnSO_4_	J	0	0.0025	0.005
FeSO_4_	K	0	0.0025	0.005
Di‐Sodium glycerol phosphate	L	0	9.5	19
NaH_2_PO_4_	M	0	20.475	40.950
Na_2_HPO_4_	N	0	21.074	42.148

**Table 1B:** Medium component screening for EPS—Various factor setting value.

Factors	Code	Setting value		
		—	0	+
Soy peptone	X_1_	0	5	10
Yeast extract	X_2_	0	5	10
Na_2_HPO_4_	X_3_	0	9.5	19
MnSO_4_	X_4_	0	0.0025	0.005
Ascorbic acid	X_5_	0	0.25	0.5

### 2.3 Kinetic Modeling of Lactic Acid Inhibition

The experiments were performed in 50 mL sterile falcon with 40 mL of medium at 42°C with stirring of 150 rpm for 5 h in a shaker incubator. The effects of product (lactic acid) at different concentrations (0, 15, 30, 45, 60, 75, 90, 105, 120, 135, and 150 g/L) on biomass production of *S. thermophilus* D4 were evaluated. pH of all experimental runs were adjusted to 7 ± 0.1 before and after sterilization of broths, adding NaOH 5 M. Initial inoculum volume was calculated to have optical density of 0.20 ± 0.05 at initial stage. All experiments were performed in duplicates. Final pH of all the experimental runs was recorded.

### 2.4 Batch and Fed‐Batch Bioreactor Experiments

The bioreactor used for the experiments was a Biostat CT plus, Sartorius Stedim (Melsungen, Germany), 2 L working volume, 3.2 L total volume. *S. thermophilus* D4 was grown at temperature of 42°C and 150 rpm, without the addition of any gas (air, oxygen, etc.). A constant pH of 7.0 was maintained via automated addition of 25% (v/v) NH_4_OH and 30% (v/v) H_2_SO_4_ (Pieretti et al. 2014). Experiments in batch mode were carried out, using the optimized medium on small 2 L scale, to better understand the consumption of carbon source and the production of lactic acid. In particular, seed stock (about 40 OD_600_) of *S. thermophilus* D4 was inoculated to 0.2 L inoculum medium in 0.25 L bottle and incubated in a rotary shaker (model Minitron, Infors, Basel, Switzerland) at 42°C and 150 rpm for 3 h. The inoculum size was 10% (v/v) and it was transferred to 2 L bioreactor to have initial optical density of 0.1 at 600 nm. The optimised media was previously ultrafiltered to eliminate any EPS contaminants from yeast extract obtaining a precise quantification of EPS produced at the end of the process. In Fed‐batch experiments, feed was added using sterile tubes when the concentration of glucose initially present in the reactor (20 g/L) was below 1 ± 0.5 g/L. Glucose concentration was restored by adding concentrated media solution (500–600 g/L) in exponential profile ranging from 8 to 10 g/L·h^−1^. During the fermentation processes samples were taken to check growth (biomass and viability), organic acids, EPS production, and glucose consumed. To check the viability, samples were serial diluted using 0.9% NaCl solution up to presumed viability dilution factor, then 10 µL was placed on M17 agar plates in triplicates. These plates were incubated at 42°C for 24 h to get viability. All experiments were performed, at least, in triplicate.

### 2.5 Quantification of Exopolysaccharides

Phenol‐sulfuric acid method was used to quantify the exopolysaccharides production (Scott and Melvin 1953; Alfano et al. 2020). The phenol‐sulphuric acid method is a simple and rapid colorimetric method to determine total carbohydrates in a sample. The method used was previously described by Alfano et al. (2020). The results reported in the table are normalized by removing the initial concentration of exopolysaccharides in the medium from the final concentration obtained.

### 2.6 Downstream Process of EPSs

EPS was recovered from the supernatant obtained by centrifugation at 6500 rpm for 30 min at 4°C at the end of the fermentation process. The supernatant was treated with 20 U/L protease from *Aspergillus oryzae* (Sigma‐Aldrich, Missouri, USA) at room temperature for 1 h and then ultrafiltered on 10 kDa polyethersulfone membranes (GE Healthcare, Illinois, USA) with 0.1 m^2^ of filtration area. Tangential flow filtration was performed on a Sartoflow alpha (Sartorius Stedim, Gottingen, Germany) system connected with a thermostatic bath that kept a constant temperature of about 20°C–25°C. The retentate was concentrated to about 1/10th of the initial volume and washed with 3 volumes of milliQ water to remove salts and low molecular weight molecules still attached to the membrane. The retentate from the ultrafiltration step was then precipitated with 2 volumes of a 1:1 (v/v) ethanol:acetone solution, after adjusting the conductivity to 14 mS/cm, and dried in a vacuum oven at 40°C over‐night. The obtained powder was then suspended at a concentration of 30 g/L in milliQ water and filtered on velapad 60 filtration system (PALL Corporation, Milan, Italy) with Pall Activated Carbon (AKS5) depth filter (Pall Corporation, Fribourg, Switzerland). The solution was precipitated with 2 volumes of a 1:1 (v/v) ethanol:acetone solution, and the pellet was dried as described above. To further increase the purity of the EPS from small molecules and salts, a step of desalting by chromatography was performed. Chromatographic system (ÄKTA Pure, Cytiva, Fålhagen, Uppsala County, Sweden), equipped with two piston pumps, an UV detector, a pH meter, a conductivity cell, a fraction collector (F‐9‐R, Cytiva, Fålhagen, Uppsala County, Sweden) all connected with a specific software (Unicorn 7.8, Cytiva, Fålhagen, Uppsala County, Sweden). The sample was injected several times on a desalting column (HiPrep™ 26/10 Desalting; GE Healthcare, Italy) and eluted in isocratic conditions with milliQ water) at pH 7.1 and at a flow rate of 2.0 mL∙min^−1^, detecting the absorbance at 215 nm. EPS containing fractions were collected, pooled, frozen at −80°C for 3 h following which freeze‐drying was performed for sublimation at −90°C at a chamber pressure of 1 mbar for 18 h followed by a secondary drying for 2 h at 0.01 mbar using a bench scale freeze dryer (Beta 2‐8 LSC plus; Christ, Gefriertrocknungsanlagen, Germany).

### 2.7 EPS Characterization

For analysis of monosaccharides composition of purified EPS, samples (20 mg/mL powder) were hydrolysed in HCl 5 M for 6 h at 100°C. Diluted and neutralised samples were analysed on a HPLC (UHPLC Dionex Ultimate 3000; Thermofisher) on a Alltech IOA‐2000 column (500 mm × 6.5 mm ID) at a flow rate of 0.6 ml/min. The mobile phase consisted of 0.1% H_2_SO_4_ in H_2_O v/v. Peak areas were evaluated through the Thermofisher chromeleon software and quantified using external standard calibration curves. Standard concentrations were linear from 10 to 0.156 mg/ml for glucose, galactose, rhamnose, and glucosamine.

### 2.8 Molecular Weight Determination by SEC‐TDA

To investigate the molecular weight of EPS produced by fed‐batch processes, a high‐performance size exclusion chromatographic system (Malvern, UK) was used. The system was equipped with a triple detector array module including a refractive index detector (RI), a four‐bridge viscosimeter (VIS), and a laser detector (LS) made of a right‐angle light scattering (RALS) detector and a low‐angle light scattering (LALS). The operation description was reported in previous papers (D'ambrosio et al. 2020 and Mohammed Sabbah et al. 2020). In particular, RALS (Right Angle Light Scattering—90°) detector provides the absolute molecular weight of proteins and small polymers, LALS (Low Angle Light Scattering—7°) detector directly measures the absolute molecular weight of polymers without extrapolation. The molecular size, that is, hydrodynamic diameter and radius of gyration Rg, are obtained by the RALS and LALS detectors in combination with the viscometer. The viscosity detector measures the intrinsic viscosity of polymers and proteins and provides structural and conformational information in any solvent. The Refractive Index detector measures the concentration of the samples.

### 2.9 In Vitro Wound Healing Assay

For the scratch wound assay, Caco‐2 cells were differentiated over 21 days and plated at a density of 3·10^5^ cells/well onto a 24‐well plate and cultured overnight in 10% FBS‐DMEM at 37◦C and 5.0% CO_2_. Once 100% confluence was reached, horizontal scratches were created in the confluent monolayer using a sterile tip (*Ø* = 0.1 mm), cellular debris was removed with sterile PBS before adding treatments. Specifically, cells were stimulated with 0.1 mg/mL of EPS‐derived *S. thermophilus* in triplicate. The in vitro wound healing assay was monitored by time‐lapse video microscopy for the entire experiment (TLVM) (OKOLAB, Naples, Italy). The instrument was assembled with an inverted microscope (AxioVision200; Zeiss Axiovert 200, Germany), a CCD‐gray camera (ORCA ER; Hamamatsu Photonics, Hamamatsu City, Japan) and a motorized incubator to maintain the general conditions of cell culture (37° C, 5% CO_2_ in humidified air); the ZEN 3.7 software allowed us not only to follow the entire process (40 h) but also to quantify the closure rate, calculated as [(Area *t*_0_–Area *t*)/Area *t*_0_] × 100, directly from the software. For each well, a minimum of 5 fields of view were used to derive wound closure (%) versus time curves, thus ensuring statistical significance of the experiment.

## 3 Results

### 3.1 Small Scale Experiments: Media Optimization Using Design of Experiment

**Figure 1:**
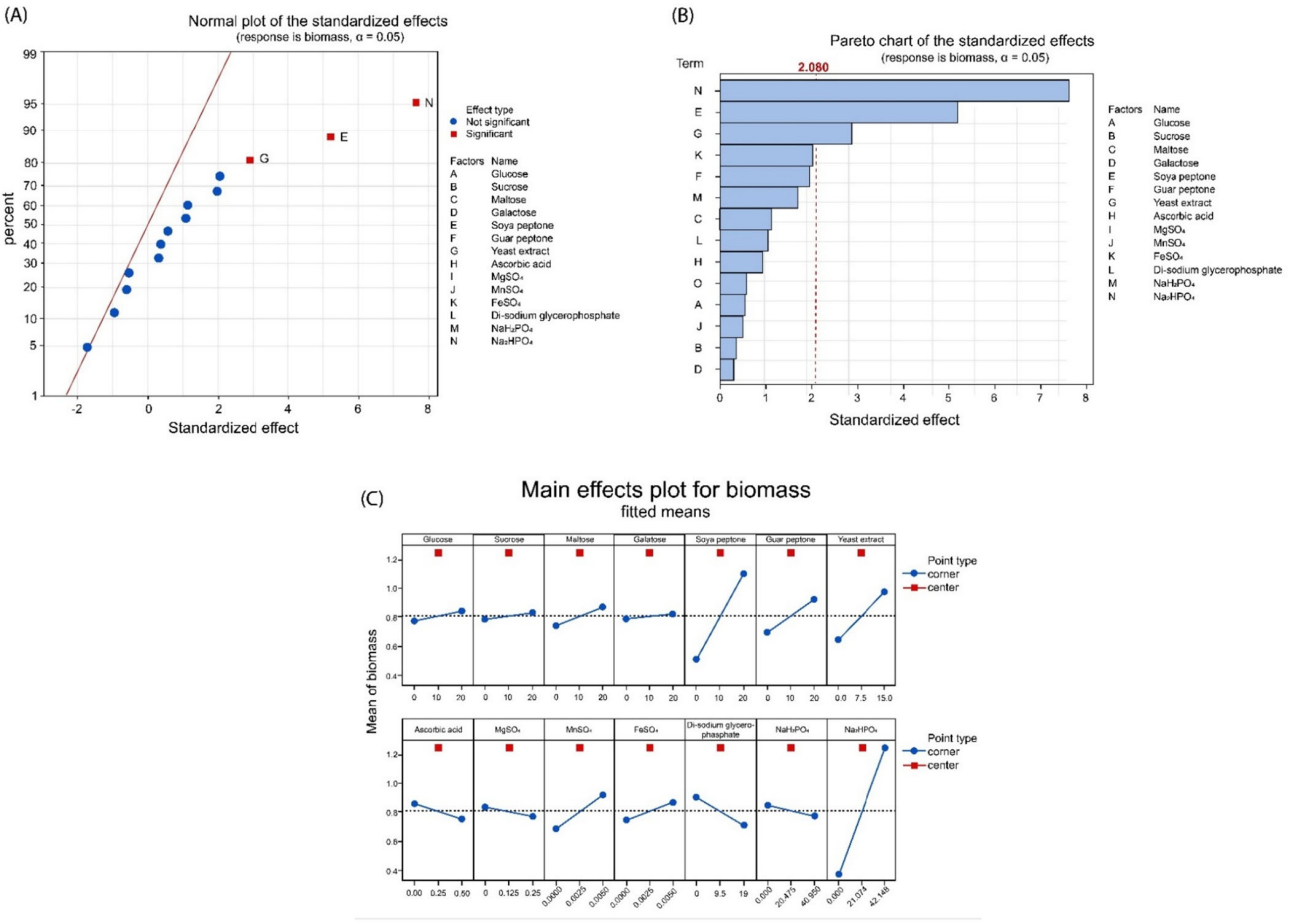
(A). Normal plot of the standardized effects for biomass production; (B) Pareto chart of the standardized effects for biomass production; (C) main effects plot for biomass production.

To identify the effect of some compounds, both positive and negative, on the growth of the microorganism and to determine the number of experiments to be carried out to obtain accurate results a Plackett–Burman screening was conducted using Minitab software. To make the experiments feasible and gain a clear idea about which component is positively or negatively correlated to the biomass titer, 37 runs were considered sufficient to perform the screening of these 14 factors. Table 1B and Figure 1 report the effect of the components on biomass yield.

Equation (1) is a regression equation derived from model used for estimation of biomass

$$\begin{array}{l} \text{Biomass}\;=-0.287+0.00321\;\left(\text{glucose}\right)+0.00206\left(\text{sucrose}\right)+0.00635\left(\text{maltose}\right)\\ +0.00168\left(\text{galactose}\right)+0.02935\;\left(\text{soy}\;\text{peptone}\right)+0.01109\left(\text{guar}\;\text{peptone}\right)\\ +0.02179\left(\text{yeastextract}\right)-\;0.213\;\left(\text{ascorbic}\;\text{acid}\right)-0.230\;\left(\text{magnesium}\;\text{sulphate}\right)+46.2\;\left(\text{manganese}\;\text{sulphate}\right)\\ +24.1\;\left(\text{ferrous}\;\text{sulphate}\right)-0.01012\left(\text{di}-\text{sodium}\;\text{glycerophosphate}\right)+0.02100\;\left(\text{Sodium}\;\text{dihydrogen}\;\text{phosphate}\right)\\ -0.00160\left(\text{disodium}\;\text{hydrogen}\;\text{phosphate}\right)+0.433\;\text{central}\;\text{point} \end{array}$$

The model obtained from the DoE experiment is more than 84% accurate with alpha value of 0.05 (Table 2). The moderate accuracy of model was expected as 14 experimental factors require more than 256 experimental runs to get 100% resolution. In just 37 experimental runs the resulting 84% accuracy is acceptable and the model provides sufficient confidence to screen‐out components from the reference media. The normal plot, pareto chart, and main effect plots of standardized effects indicate Na_2_HPO_4_ with effect value of 7.6, soya peptone with effect value of 5.2, and yeast extract with effect value of 2.9. This is well beyond the significant mark of 2.08 effect value. The effects of all these 3 components is positive on biomass production, while ascorbic acid, MgSO_4_, di‐sodium glycerophosphate, and NaH_2_PO_4_ a negative effect on biomass production was found. Others do have positive effect values but not significant enough to be used for higher biomass production.

**Table 2:** Biomass screening model accuracy.

S	0.338029
R‐seq	84.07%
R‐seq Adjusted	72.70%
α	0.05

### 3.2 Small Scale Experiments: Comparison Between Optimized and Reference Media (M17)

Small‐scale experiments, conducted using Plackett–Burman screening, were used to identify the effect of some compounds on biomass production by determining the optimal concentrations of the culture medium components. The newly developed media was tested against M17 as reference medium. The results of this experimental study were reported in Table 3. The growth of microorganism (OD_600_) in optimized media was found to be 4.9 times higher than M17 media. Lactic acid production was 6.68‐fold higher and dry cell weight was multiple of 4.8 factor higher. The EPS produced was 13 times higher in optimized media.

**Table 3:** comparison between reference medium and optimized medium.

	M17	Optimize medium
OD_600_	1.60	7.90
Lactic acid produced (g/L)	2.50	16.70
Yield (lactic acid produced/substrate consumed)	0.5	0.83
Dry cell weight (g/L)	0.50	2.40
EPS (g/L)	0.14	1.81

**Figure 2:**
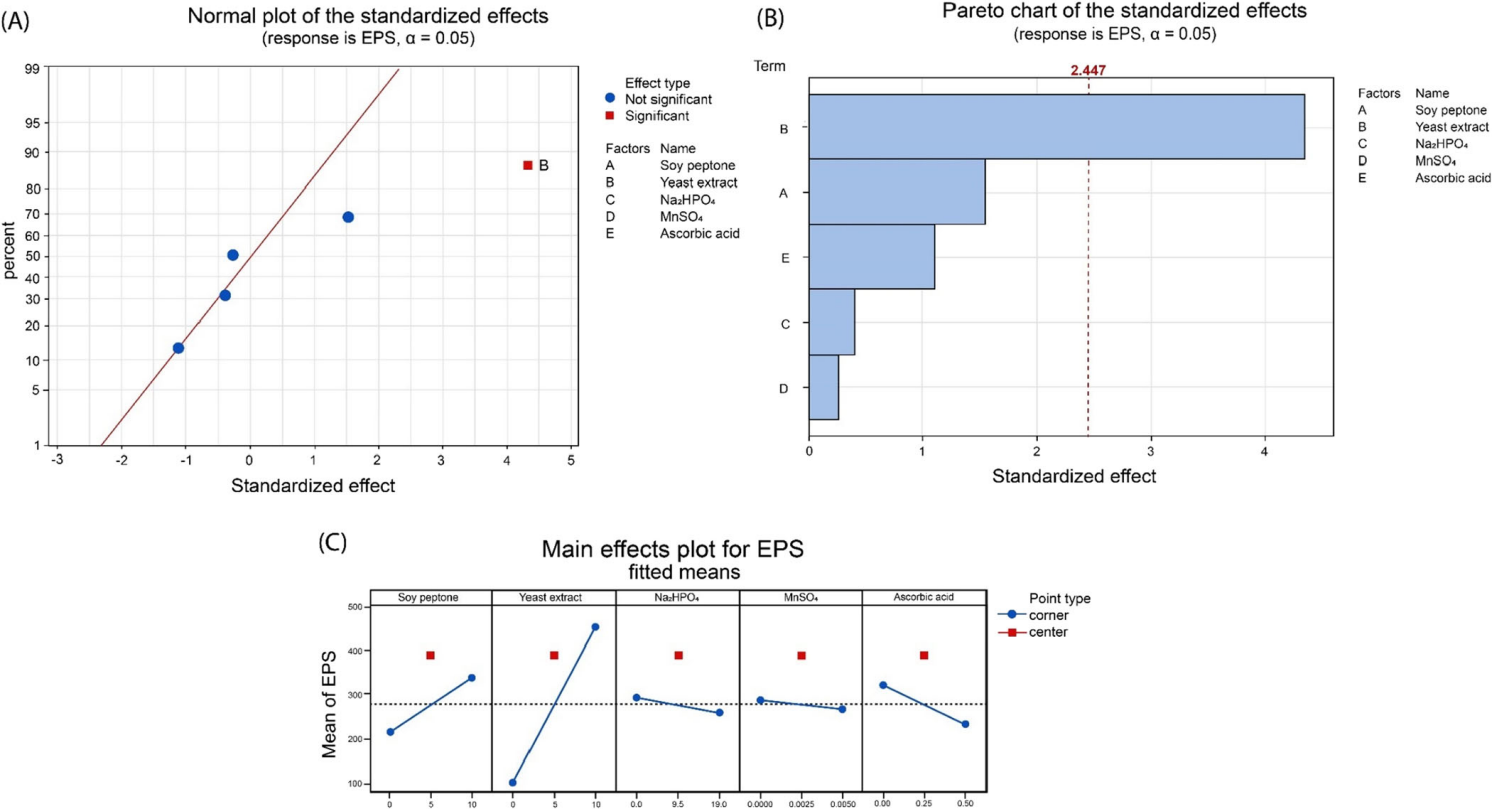
(A) Normal plot of the standardized effects for EPS production; (B) Pareto chart of the standardized effects for EPS production; (C) main effects plot for EPS production.

After optimizing media, another DoE experiments were performed using Plackett–Burman Screening method. To make understanding of EPS production clearer and to have accurate results, the number of experimental runs were kept as minimum as possible. Small number of experiments allowed us to reduce the number of chemicals fermentation media as maximum as possible and then analyses them. 13 experimental runs were found to be more than sufficient for the purpose and used only 5 factors rationally to check its effectiveness. Figure 2 shows the effect of the components on EPS production.

Equation 2: Regression equation derived from model.

$$\text{EPS}=222.6+12.52\;\text{Soy}\;\text{Peptone}+35.15\;\text{Yeast}\;\text{Extract}-1.72\;{\text{Na}}_{2}{\text{HPO}}_{4}-4282\;{\text{MnSO}}_{4}-178\;\text{Ascorbic}\;\text{acid}$$

The normal plot, pareto chart, and main effect plots of standardized effects indicates soy peptone with effect value of 4.34. This is well beyond the significant mark of 2.44 effect value. Na_2_HPO_4_, ascorbic acid, MnSO_4_, and Na_2_HPO_4_ found to have negative effect on biomass production. Soy peptone has positive effect but not significant enough to be used for higher EPS production.

### 3.3 Small Scale Experiments: Lactic Acid Tolerance

Based on previous studies conducted on *Lactococcus lactis* I7, it is essential to understand the ability of this strain to tolerate lactic acid at specific pH values and temperatures (Alfano et al. 2025). In this study, we also investigated further the correlation between the fermentation process at uncontrolled pH and that at controlled pH.

**Figure 3:**
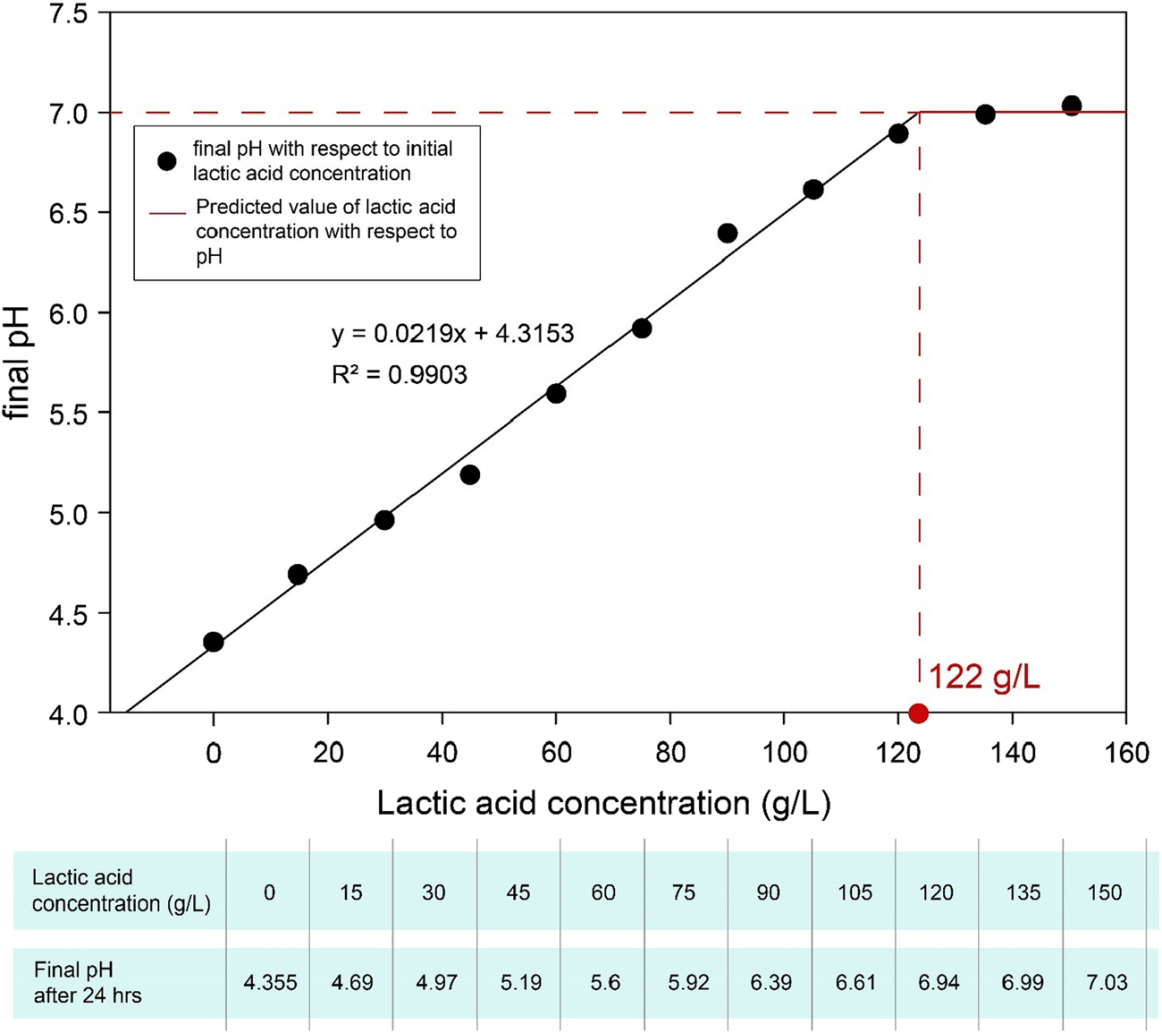
pH versus Lactic acid concentration behavior.

Final pH value measured after 24 h of fermentation was drafted against lactic acid concentration present initially in the media as shown in figure 3. The final pH indicates strain's ability to tolerate lactic acid concentration. The initial pH of *S. thermophilus* D4 in each run is 7.0. The line with equation *y* = 0.0219*x* + 4.3153 seems to touch pH 7 at lactic acid concentrations of 122 g/L.

**Figure 4:**
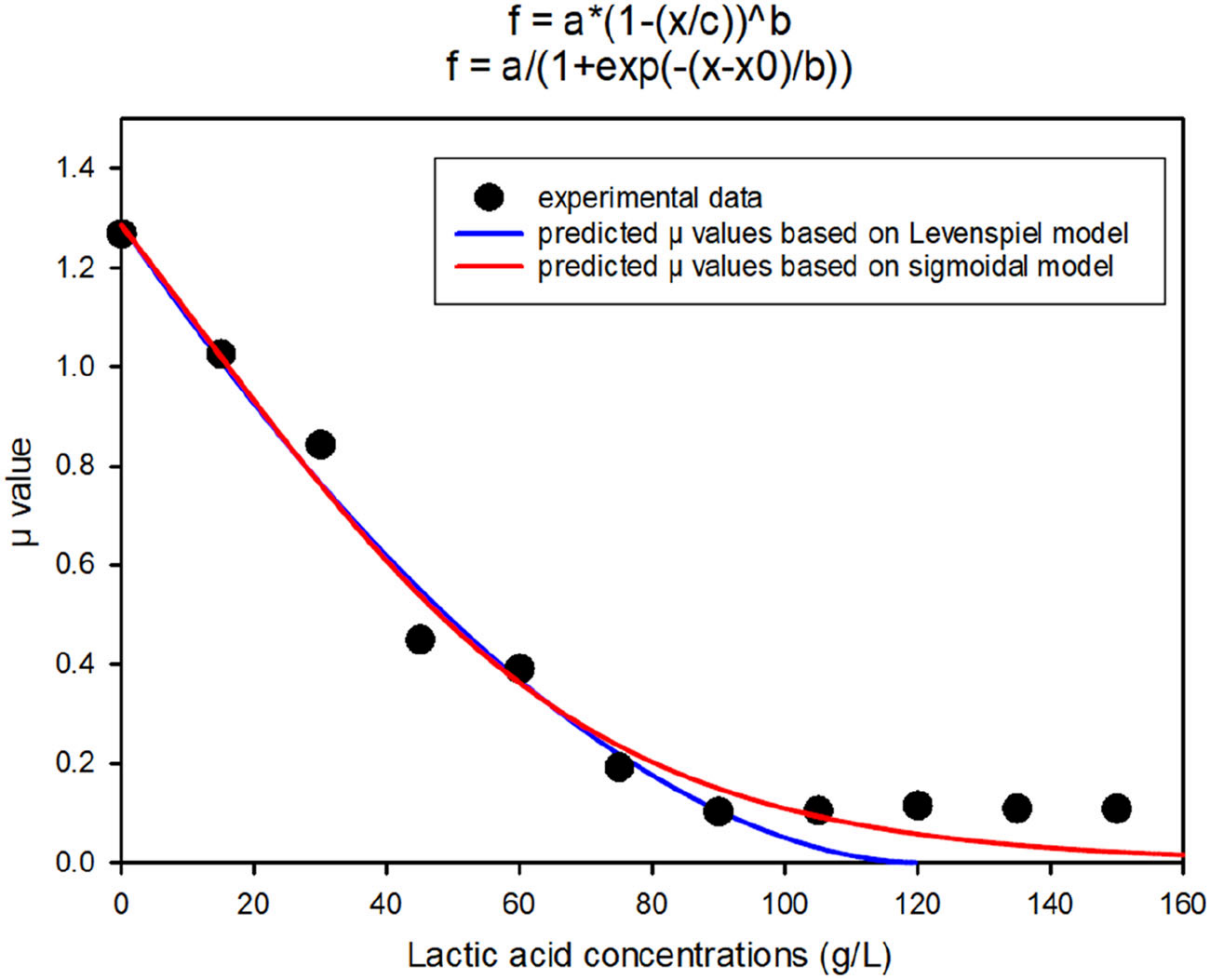
Lactic acid concentration versus *µ* value.

The data was used to get *µ* value for each concentration. The graph of lactic acid concentration and *µ* was plotted as shown in Figure 4.

Equation 3.

$$\mu =\frac{\mu_{\max}}{1+\;e^{\lambda \left(x-x_{0}\right)}}$$

Where μ is specific growth rate (h^‐1^), $\mu_{\max}$ is maximum specific growth rate (h^‐1^), λ is constant specific to strain; x is lactic acid concentration at given moment (g/L), $x_{0}$ is 12.10 g/L (Lactic acid concentration from where specific growth rate starts significant descent).

Equation 4.

$$\mu =\mu_{\max}{\left(1-\frac{x}{c}\right)}^{n}$$

Where, μ is specific growth rate (h^‐1^), $\mu_{\max}$ is maximum specific growth rate (h^‐1^), x is lactic acid concentration at given moment (g/L), c is maximum possible lactic acid concentration (g/L), n is constant specific to strain.

Equations (3) and (4) were plotted as shown in Figure 4 and their *R* ^2^ value were calculated for the accuracy (Rathore and Kapoor 2016). Both the equations have *R* ^2^ value of 0.99.

Figure 4 shows a clear decrease in growth around 60 g/L lactic acid with a reduction in the specific growth rate of about 3 times. However, growth is blocked only at 120 g/L demonstrating a very high tolerance to lactic acid and confirming the results obtained previously and shown in figure 3.

### 3.4 Bioreactor Experiments: Biomass, EPS, and Lactic Acid Production

Batch and fed‐batch processes in bioreactor were performed to verify the ability of *S. Thermophilus* D4 to produce EPS, biomass and lactic acid using optimized medium. The data obtained from batch and fed‐batch processes are reported in Table 4, and they were compared with the M17 media used as control. Fed‐batch processes increased biomass production approximately 2.5‐ and 7.5‐fold than batch processes with optimized and M17 media, respectively. EPS production increased, in fed‐batch processes, by 1.7‐ and 6‐fold compared to batch processes with optimized and control media, respectively. Viability in fed‐batch processes also increased by 28‐ and 3‐fold compared to batch processes with optimized media and control media, respectively. Similar trends were observed in lactic acid production. Optimized media were able to produce 23‐fold more lactic acid than M17 media. On the other hand, fed‐batch experiments were able to produce 5.5‐fold higher lactic acid concentrations compared to batch experiments. In all steps of process development, yield and productivity of process was observed to be increasing significantly.

**Table 4:** Resume of the results obtained in the different types of process.

Type of process	Medium	OD_600_	Biomass (g/L)	EPS (g/L)	Viability max (CFU/mL)	Lactic acid production (g/L)	Yield of the process (lactic acid produced/substrate consumed)	Productivity (g/L ∙ h)
Batch	M17 (control media)	3.0 ± 0.1	1.4 ± 0.03	0.6 ± 0.04	0.6 ± 0.1 ∙ 10^9^	5.25 ± 0.2	1.05 ± 0.04	0.24 ± 0.03
Batch	Optimized media	11.5 ± 1.5	4.3 ± 0.1	2.2 ± 0.1	5.6 ± 0.2 ∙ 10^9^	22.2 ± 0.4	1.11 ± 0.13	1.0 ± 0.15
Fed‐batch	Optimized media	48 ± 2.5	10.7 ± 0.1	3.7 ± 0.2	1.7 ± 0.1 ∙ 10^10^	121.2 ± 1.3	1.21 ± 0.15	5.5 ± 0.15

### 3.5 Molecular Weight Determination and Monosaccharide Composition of EPS

**Figure 5:**
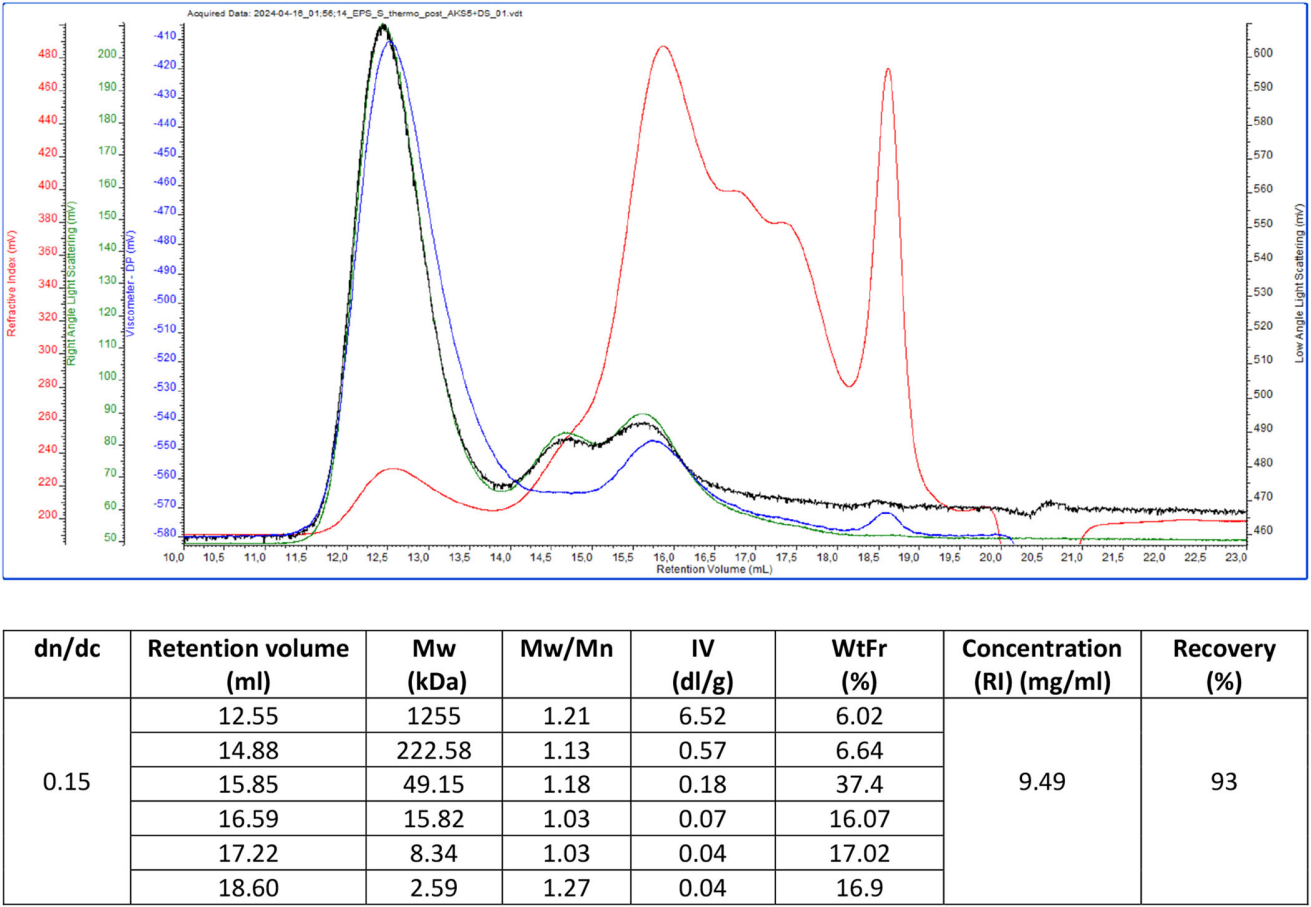
SEC‐TDA analysis and fractions characterization of the EPS produced by *S. thermophilus* D4. Retention volume (mL) on the x‐axis. On the y‐axis in red RI, in green RALS, in black LALS, in blu intrinsic viscosity.

After the purification process, the molecular weight of the polysaccharides produced by *S. thermophilus* D4 was evaluated by SEC‐TDA. The solids recovered from the broth at the end of the purification procedure were equal to 6.05 g. The concentration of polysaccharides in the sample was estimated by analyzing the sample through SEC‐TDA. The latter indicated the presence of a population of different polymers/oligosaccharides with a range size from 1250 to 2.6 kDa. In fact, the chromatogram reported in Figure 5 indicates the presence of six species in the sample derived from the fed‐batch processes. In this range, the 37.4% of the polysaccharide species had a molecular size of 49.1 kDa (Figure 5).

The solid fraction recovered at the end of the downstream process based on UF/DF and solvent precipitation, and containing the exopolysaccharide released in the culture medium during cultivation was hydrolysed using acidic conditions and analysed by U‐HPLC to obtain the main monosaccharides compositions. Results presented a major abundance of galactose and especially glucose in respect to other peaks not included in the standards calibration curve. In particular, the EPS resulted in a composition mainly of 1 glucose: 0.4 galactose. The composition found is confirmed to be common to many *S. thermophilus* strains already characterized by different authors (Navarini et al. 2001 and Säwén et al. 2010).

### 3.6 Potential of EPS in Wound Healing Experiments

**Figure 6:**
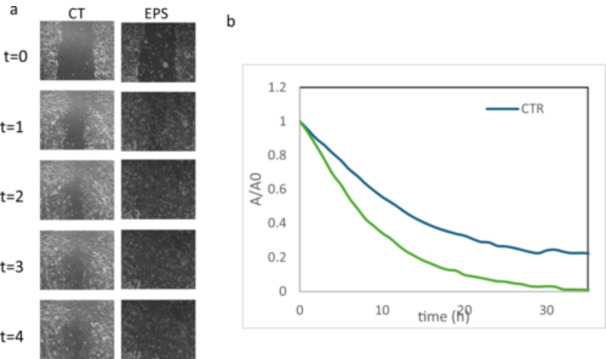
(a) Microscopic images obtained from the scratch wound healing assay in Caco‐2 cells treated with 0.1 mg/ml of EPS derived from *S. thermophilus* compared to untreated cells (CTR). A representative pictures of HaCaT monolayer after the scratch and in time course of experiment recovered by time lapse software. (b) Quantitative analysis of wound closure in the time.

To determine the effect of EPS on differentiated Caco‐2 cells (a model for physiological enterocytes), wound healing experiments using time‐lapse video microscopy were performed. The size of the scratched area was monitored for 40 h to assess the eventually occurring beneficial potential of EPS derived from *S. thermophilus*. After analyzing images obtained from the time‐lapse microscopy and comparing them to the untreated condition (CTR) (figure 6), EPS treatment exerted a significant wound healing activity. Specifically, the wound closure in cells treated with of EPS derived from *S. thermophilus* was almost completed with a residual scratched area of about 5% compared to the remaining 35% area in the untreated cells (CTR). Results of wound closure are shown in Table 5.

**Table 5:** Effect of EPS treatment on wound closure: time for reaching 20%–60%–80% to wound closure *p < 0.05 versus CTR.

Sample	Time to 20% wound closure (h)	Time to 60% wound closure (h)	Time to 80% wound closure (h)
CTR	8.8 ± 0.6	14.5 ± 0.9	37.7 ± 5.0
EPS *S. Thermophilus* D4	5.3 ± 1.0*	8.6 ± 2.0*	14.4 ± 4.0

EPS from *S. thermophilus* have shown promising effects in accelerating wound healing. According to the *A*/*A*_0_ graph, showing the kinetic of scratch closure upon enterocytes migration, the presence of EPS significantly improved wound closure respect CTR, with a notable reduction in the time required for healing, reaching nearly full closure within approximately 25 h. The analysis of the wound healing process in terms of closure percentages (specifically 20%, 60%, and 80%) as reported in Table 5 demonstrated that EPS significantly accelerated the repair mechanism respect the CTR.

## 4 Discussions

High production of probiotic biomass and exopolysaccharides requires a cost‐effective production method and nutrient media optimization (Chang and Liew 2013). For example, the biomass production of probiotic strain and the metabolites of interest can be improved by optimizing medium components such as carbon and nitrogen sources and salts concentrations (Aristimuño Ficoseco et al. 2023). For these reasons DoE experiments were performed to obtain optimal media and different types of bioreactor processes of *S. thermophilus* D4. The *S. thermophilus* strain typically used in food processing have been reported to produce EPS about 200‐600 mg/L in M17 media (Padmanabhan et al. 2020 and Wa et al. 2022). The increase in yield and productivity reduced the costs making the process more competitive for further commercial applications. This paper focuses on the importance of vegan‐grade media for starter culture and types of processes for metabolites production (exopolysaccharides) and its uses.

The model obtained from the DoE experiment is more than 84% accurate with alpha value of 0.05 (Table 2). This model follows the various indications reported by Ghose A. and collaborators to obtain reproducible results by significantly reducing the number of experiments (Ghose and Ravindran 2020).

The results of the experimental tests clearly indicate that *S. thermophilus* D4 needs at least one additional nitrogen source along with yeast extract in the medium to grow (figure 1). As reported by Ricciardi A., Oluwayemisi F. R., and collaborators yeast extract and peptone are the main energy source for EPS production (Ricciardi et al. 2002; Oluwayemisiet al. 2020). Vegan grade nitrogen source soy peptone is performing better than guar peptone; thus, it was decided to only proceed with soy peptone in combination with yeast extract, because it offers wider range of nutrients, provides higher nitrogen source and it has a significant and positive effect on biomass production (Figure 1A,B). The effect of di‐sodium hydrogen phosphate is found to be very strong that all other salts and sugar sources were not relevant (Dyaee and Luti 2019). In screening experiments, the influence of sodium and phosphate in the growth of *S. thermophilus* to increase biomass and exopolysaccharides production was evaluated. In fact, we compared di‐sodium hydrogen phosphate with di‐sodium glycerol phosphate and sodium di‐hydrogen phosphate. Only di‐sodium hydrogen phosphate indicated positive effects respect to other sources of phosphate and sodium ion. The positive effect of disodium hydrogen phosphate has never been reported before for higher biomass production of lactic acid bacteria. Its ability to act as a buffer is known, helping to stabilize the pH of the growing medium, an essential factor for optimal nutrient absorption and to support microbial activity. But, it is widely reported for other microorganisms and algae like *Spirulina platensis*, *Burkholderia cepacian*, and *Pseudomonas aeruginosa* (Çelekli et al. 2009; Xu et al. 2015; Wang et al. 2021). The media component's effect was also verified for EPS production. Ascorbic acid and manganese sulphate was also tested for EPS production based on previous literature (Lee et al. 2010; Liu et al. 2018; Ulmer et al. 2022; Kapse et al. 2024, and Zanzan et al. 2024).

The results, which are in line with literature, indicate only yeast extract giving significant EPS production compared to other 4 factors tested in the screening experiments. The ratio between yeast extract and soy peptone was kept being 3:4 in weight. This was due to soy peptone's ability to contribute tobiomass production as well as to EPS production. The strain *S. thermophilus* D4 has shown ability to produce EPS at various concentrations in a huge range between 3 and 1100 mg/L, depending on growth, media and conditions. As shown in table 3, *S. thermophilus* D4 produced 144 mg/L of EPS in M17 media in bottle, that is very similar to previously reported literature results (Xu et al. 2021; Karadeniz et al. 2021). After successful DoE, EPS production was 1807 mg/L in optimized media, that is, 12.5‐fold increase. This is 40% more than value of EPS production from *S. thermophilus* previously reported (Wu et al. 2014).

Simplified media contains glucose, soy peptone, yeast extract, and di‐sodium hydrogen phosphate, with 1:1.5 C:N ratio. Glucose was preferred to maltose because the results obtained do not show substantial differences but it has a greater industrial demand due to its easy availability and reduced cost (Yankov 2022; Konzock and Nielsen 2024). As per the model, replacement of glucose with maltose will not create any significant difference in biomass production. To minimize noise produced from various carbon sources, biomass optimization and EPS optimization experiments were performed separately. So, in biomass optimization four different carbon sources were tested, but in EPS optimization experiments only glucose (10 g/L) was used. The concentrations of these components were finalized using literature and carbon/nitrogen ratio. No interaction between two components was observed during screening experiments. The optimised vegan grade medium was compared with M17 medium. The optimised vegan grade medium increased the production of biomass and lactic acid of 4.8‐ and 6.7‐fold, compared to M17 medium in small‐scale bottle experiments, respectively (Table 3). It was expected that the pH‐controlled experiments in the bioreactors would increase the amount of biomass since in the bottle the growth was stopped due to the low pH of 4.3 and not due to the exhaustion of the carbon source, still present after 24 h of growth. Lactic acid inhibition studies at small scale were performed to understand ability of *S. thermophilus* D4 to tolerate lactic acid concentration at given pH, obtaining important information for the optimization of fed‐batch profile. The results based on final pH values after fermentation at different lactic acid concentrations indicated the maximum lactic acid concentrations at pH 7 was 122 g/L. Thus, we designed a feed profile in such a way that, it will add enough substrates to reach 122 g/L lactic acid without residual carbon sources.

Since biomass and EPS production could be linked to lactic acid production, it is important to understand design space of the fermentation process associated with strain's ability. Lactic acid production is itself linked with process temperature, pH and lactic acid concentration present in the media (Abedi and Hashemi 2020). Due to resource restraints, effect of temperature on process pH and lactic acid concentrations in media were not studied. Keeping only co‐relation of pH and lactic acid concentrations in experimental study lactic acid inhibition studies at small scale were performed to understand ability of *S.thermophilus* D4 to tolerate lactic acid concentration at given pH, obtaining important information for the optimization of fed‐batch profile. The results were exactly as expected. All the fed batch experiments produced lactic acid concentrations around 122 g/L, as expected. Equations (3) and (4) produced almost similar *R* ^2^ values. Thus, fortifying the possibility of sigmoidal models to be more accurate. The equation indicated the possible µmax value to be 2.14. But interestingly the values of equation helped us to predict precise µmax value for the bioreactor experiments. This highlighted that Equation (3) was more reliable than equation 4 in predicting the value of µmax, while Equation (4) was shown to be more effective in predicting the strain tolerance to lactic acid concentration. The new sigmoidal equation seems to make more sense as if typical biomass production growth follows sigmoidal curve, then product inhibition in absence of substrate inhibition should also follow sigmoidal curve. The new sigmoidal curve is the outcome of experimental results plotted using modern software to achieve the highest possible accuracy.

Considering the data obtained in the lactic acid inhibition tests, an exponential feeding profile was designed to maximize yields and productivity. Thanks to the fed‐batch process with a suitable time profile and optimized medium, a 15% increase in yield and 96% in productivity was obtained compared to the control process (batch with M17 medium). The yield coefficient (lactic acid produced on glucose consumption) was higher than 1 g/g probably due to the contemporary uptake of the amino acids (i.e., peptides/proteins) present in the nitrogen sources (yeast extract and soy peptone) that increase the lactic acid production. At the end of the experimental studies, lactic acid production and biomass production were found to be maximized with the highest possible yield successfully with keeping design space in consideration. EPSs production is three times higher than the maximum EPSs production reported so far in literature by any other *S. thermophilus*, but lower than *Levilactobacillus brevis* and in line with *Leuconostoc mesenteroides subsp. Mesenteroides* (Ramos et al. 2023). Furthermore, EPS from *S. thermophilus* has shown significant potential in reducing intestinal inflammation (Chen et al. 2019). However, its regenerative properties, particularly in skin tissue, have not yet been studied. In our model EPS from *S. thermophilus* has been shown to have an action on in vitro gut repair mechanism similar to that reported by EPS of other strains (Lee et al. 2021; Zaghloul and Ibrahim 2022; Xu et al. 2024).

## 5 Conclusion

In this study work, an integrated bioprocess development along with methodical and calculative Quality by Design approach has been designed for the newly isolated *S. thermophilus* D4 strain from buffalo natural whey starters. Optimization of culture media with DoE, development of fermentation processes, purification and recovery of EPSs tested on cells were performed.

A brief component screening using Plackett–Burman screening model showed that most of the salts and trace elements generally present in M17 are not affecting biomass production, thus a simplified media preparation, with glucose, yeast extract, soy peptone, di‐sodium hydrogen phosphate has been proposed. The concentrations of used components used in this media are decided using literature‐based rational. This was followed by another screening to check effect of each component on EPS production to further improve EPS production was done using Plackett–Burman Screening.

The results showed an increase of biomass and EPSs production using fed‐batch processes. Kinetic modeling was used to estimate the maximum possible lactic acid tolerance providing very important value of substrate concentration in feed media to maximize process yield and productivity. Overall, design of experiments along with literature and experience‐based rational were applied to quickly get very efficient vegan‐grade optimized media for biomass and exopolysaccharide production, which was used for process development along with kinetic modelling to optimize productivity and yield. Use of design of experiments and kinetic modelling provides approach integrated with Quality by Design.

## Author Contributions

**Alberto Alfano:** conceptualization of the project experimental plan, writing – original draft and editing. **Alberto Alfano** and **Darshankumar Parecha:** performed bioreactor experiments. **Darshankumar Parecha:** performed design of experiments, data analysis, and numerical modelling, viability studies. **Donatella Cimini:** and **Sergio D'ambrosio:** downstream process experiments and characterization of EPS. **Maria d'Agostino:** performed experiments on cell. **Chiara Schiraldi:** visualized project, project administration, writing original draft, review and editing. **Chiara Schiraldi:** performed funding acquisition, and supervision for the experimental activities.

## Ethics Statement

This article does not contain any studies with human participants or animals performed by any of the authors.

## Conflicts of Interest

The authors declare no conflicts of interest.

## Data Availability

Data is contained within the article. The author declare that the data supporting the findings of this study are available within the paper and its supplementary information files. Data sharing not applicable to this article as no datasets were generated or analysed during the current study.
